# Development and validation of a machine learning–based risk prediction model for non-suicidal self-injury in adolescents

**DOI:** 10.3389/fpsyt.2026.1837161

**Published:** 2026-05-13

**Authors:** Yujun Zhao, Qian Wang, Wei Liu

**Affiliations:** 1 Department of Affective Disorders, The Third Honorable Military Hospital of Hebei Province, Baoding, Hebei, China; 2 Department of Clinical Psychology II, Hebei Provincial Mental Health Center and The Sixth Clinical Medical College of Hebei University, Baoding, Hebei, China

**Keywords:** adolescents, machine learning, non-suicidal self-injury (NSSI), suicide-related behaviors, support vector machine (SVM)

## Abstract

**Background:**

Non-suicidal self-injury (NSSI) among adolescents has long been an important social issue. This study aims to construct a predictive model for NSSI based on machine learning models.

**Methods:**

A retrospective cohort study design was adopted, including 588 adolescent patients who received psychological and psychiatric assessments. The occurrence of NSSI behavior was used as the outcome variable. Candidate predictors including demographic characteristics, psychological and emotional status, behavioral characteristics, and peer support were collected. The dataset was randomly divided into a training set and a test set at a ratio of 7:3. By comparing the performance of four machine learning models—multiple logistic regression (MLR), random forest (RF), support vector machine (SVM), and eXtreme Gradient Boosting (XGBoost)—at different time points (T1, T2, T3) using area under the curve (AUC), accuracy, precision, recall, and F1 score, the optimal model was selected. The Shapley additive explanations (SHAP) method was further used to conduct interpretability analysis for the optimal model.

**Results:**

The incidence rates of NSSI at T1, T2, and T3 were approximately 24%, 23%, and 22%, respectively. The SVM model demonstrated superior discrimination ability and stability in predicting the risk of NSSI among adolescents, with AUC values all greater than 0.75 and recall and F1 scores both higher than 0.7. SHAP analyses at all three time points consistently showed that suicide-related ideation and behaviors, school bullying, and depressive status had high contributions to the prediction of NSSI risk.

**Conclusion:**

The support vector machine model performed best in predicting NSSI among adolescents. Suicide-related behaviors are important predictors of NSSI. The findings of this study help improve the early identification of adolescents at high risk of NSSI and provide evidence for developing targeted prevention and intervention strategies.

## 1 Introduction

Non-suicidal self-injury (NSSI) refers to behaviors in which individuals deliberately cause damage to the surface of their bodies without suicidal intent, and such behaviors are not socially or culturally sanctioned (1). Studies have shown that NSSI is prevalent among adolescents, both in community and clinical settings, with a lifetime prevalence ranging from 17% to 60% (2). Non-suicidal self-injury is not only an important psychological and behavioral problem but is also considered a significant risk factor for various mental disorders and adverse psychological outcomes. Frequent NSSI can significantly increase the risk of suicide and is closely associated with the occurrence of depressive disorders (3, 4), anxiety disorders (5, 6), borderline personality disorder (7, 8), and substance use disorders (9). Therefore, the early identification of individuals at high risk of NSSI and the implementation of effective intervention measures are of great public health significance for promoting adolescent mental health.

In recent years, with the development of data science, machine learning methods have been increasingly applied in the fields of mental health and public health research, particularly in predictive modeling (10). Compared with traditional statistical models, machine learning relies on patients’ clinical testing data or monitoring data, can handle complex high-dimensional data, and identify potential nonlinear relationships among variables, thereby demonstrating higher accuracy in disease risk prediction (11). Previous studies have shown that the risk factors for NSSI are multifaceted. Among them, psychological factors are widely recognized as important correlates, including anxiety, depression, and difficulties in emotion regulation (12). Environmental and interpersonal factors are also important, with experiences such as bullying victimization, peer rejection, and adverse interpersonal relationships being significantly associated with an increased risk of NSSI (13). Adverse childhood experiences, such as poor parent–child relationships and insufficient family support, have also been shown to increase vulnerability to NSSI (14). In addition, insufficient sleep has been identified as a risk factor for NSSI (15). However, although several studies have applied machine learning to predict NSSI among adolescents, most of them are based on cross-sectional data. For example, Ben et al. predicted risk factors for NSSI among adolescent girls through a cross-sectional study (16). Similarly, Yunling et al. used machine learning methods in a cross-sectional study to identify risk factors for NSSI among adolescents in northwest China (17).

These studies are limited in their ability to reveal temporal variations in risk factors. Therefore, the present study constructed a progressive prediction framework with multiple time windows and compared the performance of several machine learning models in predicting the risk of NSSI among adolescents. By integrating longitudinal data analysis with interpretable machine learning methods, this study aims to improve the early identification of adolescents at high risk of NSSI and provide scientific evidence for developing targeted prevention and intervention strategies.

## 2 Materials and methods

### 2.1 Study design

This study retrospectively included adolescents who attended the psychiatric department of our hospital between July 2021 and July 2023, which represents the baseline enrollment window. The inclusion criteria were as follows: (1) age between 12 and 18 years; (2) ability to understand and independently complete the relevant psychological assessment scales or complete the questionnaires with guidance from researchers; (3) availability of complete follow-up outcome data; 4) During the follow-up period, none of the included participants received systematic or standardized interventions specifically targeting NSSI. The exclusion criteria were: (1) loss of outcome data at any follow-up stage; (2) inability to continue participating in the study due to suicidal behavior, refusal to eat, or coma; (3) presence of severe physical illnesses such as malignant tumors, advanced-stage cancer, or severe hepatic and renal dysfunction; and (4) comorbid cognitive impairment that prevented the participant from completing the questionnaires independently or with guidance from researchers. After applying the inclusion and exclusion criteria, a total of 588 participants were finally included in the study.

### 2.2 Data collection

Demographic information, including age, sex, and grade, was collected through a structured self-report questionnaire. At baseline (T1), information on whether adolescents had suicidal ideation, suicide plans, suicide attempts, experiences of school bullying, and truancy behaviors was collected, all of which were treated as binary variables. Depressive symptoms were assessed using the Center for Epidemiologic Studies Depression Scale (CES-D) (18); anxiety symptoms were measured using the Screen for Child Anxiety Related Emotional Disorders (SCARED) (19, 20) the severity of internet addiction was evaluated using the Young Internet Addiction Test (IAT) (21); and sleep quality was assessed using the Pittsburgh Sleep Quality Index (PSQI) (22). Perceived social support was measured using the Multidimensional Scale of Perceived Social Support (MSPSS) (23, 24). Emotional and behavioral problems were assessed using the Strengths and Difficulties Questionnaire (SDQ) (25). All of these scales have demonstrated good reliability and validity and have been validated in China. Suicide-related behaviors, school bullying, truancy behaviors, and all the above psychological and behavioral scales were reassessed and collected during the follow-up periods at 6 months (T2) and 1 year (T3). Using variables at baseline (T1) as predictors to estimate the occurrence of NSSI at T1, T2, and T3.

### 2.3 Measurements

The CES-D consists of 20 items, each rated on a 4-point scale, with a total score of 60. Higher scores indicate more severe depressive symptoms. Scores of 0–15 indicate no or minimal symptoms, 16–21 indicate possible depressive symptoms (requiring further evaluation), and ≥22 indicate significant depressive symptoms.

The SCARED includes 41 items, each rated on a 3-point scale (0–2), with a total score of 82. Higher scores indicate more severe anxiety symptoms. The commonly used cut-off values are ≥23 for child self-report and ≥27 for parent report.

The IAT consists of 20 items rated on a 5-point scale (1–5), with a total score of 100. Higher scores indicate a greater degree of internet addiction.

The PSQI comprises 7 components, each scored from 0 to 3, with a total score of 21. Higher scores indicate poorer sleep quality, and a total score >5 suggests sleep disturbances.

The friends subscale of the MSPSS includes 4 items rated on a 7-point scale, with a total score of 28. Higher scores indicate greater perceived social support from friends.

The SDQ consists of 25 items rated on a 3-point scale (0–2), with a total difficulties score of 40 (prosocial behavior is scored separately). Higher scores indicate greater emotional and behavioral difficulties. Scores of 0–15 indicate low need, 16–19 indicate some need, and ≥20 indicate high need.

All scales demonstrated good internal consistency in the current sample, with Cronbach’s α coefficients ≥ 0.75.

### 2.4 Outcome measures

In this study, NSSI was assessed using the Deliberate Self-Harm Inventory (DSHI) (26, 27). This scale includes various forms of self-injurious behaviors, such as cutting, burning, scratching, biting, stabbing oneself with sharp objects, hitting the body against objects, punching oneself, and other self-harm behaviors. Participants who reported any form of self-injurious behavior listed in the DSHI within the past year were classified into the NSSI group; otherwise, they were classified into the non-NSSI group.

### 2.5 Machine learning

Multiple machine learning methods were used for model construction and analysis in this study, including Multiple Logistic Regression (MLR), Support Vector Machine (SVM), Random Forest (RF), and Extreme Gradient Boosting (XGBoost) models. First, the dataset was randomly divided into a training set and a test set at a ratio of 7:3. The training set was used for model development, while the test set was used solely for performance evaluation and was not involved in any model training or hyperparameter optimization. A nested cross-validation framework was employed to evaluate model performance. Specifically, the training set was stratified into five folds based on the outcome variable. In each outer fold, one fold was used as a validation set, and the remaining four folds were used for training. To achieve optimal model performance, for the XGBoost, grid search was conducted over a predefined parameter space, including maximum tree depth (max_depth), learning rate (eta), subsample ratio (subsample), and feature sampling ratio (colsample_bytree). For the random forest model, key parameters such as the number of randomly selected features (mtry) were optimized via grid search, and a sufficient number of trees was set to ensure model stability. For the support vector machine model with a radial basis function kernel, hyperparameters including the penalty parameter (cost) and kernel parameter (gamma) were tuned. Bootstrap resampling was applied to estimate the mean values and 95% confidence intervals of accuracy, precision, recall, and F1 score. Receiver operating characteristic (ROC) curves were plotted on the test set, and accuracy, precision, recall, and F1 score were calculated to assess the model’s generalization performance, thereby providing internal validation. We implemented a sampling-based Shapley value approximation using the iml package in R. SHAP values were computed based on a Monte Carlo approximation of Shapley values, in which weighted linear regression over sampled feature coalitions was used to estimate the marginal contribution of each feature to individual predictions. The training dataset was used as the background data. Model performance was evaluated using the cross-entropy loss function as the criterion, and global feature importance was assessed by aggregating each feature’s contribution to changes in prediction error. Finally, features were ranked according to their average contribution and visualized accordingly. Given the sample size (n = 588) and the number of predictors (n = 11), this study represents a low-dimensional modeling problem and is suitable for machine learning analyses using methods such as Random Forest and XGBoost.

### 2.6 Statistical analysis

Continuous variables were expressed as median (minimum–maximum) and analyzed using the independent samples t-test or the Mann–Whitney U test. Categorical variables were expressed as frequency (percentage) and analyzed using the chi-square test or Fisher’s exact test. All statistical analyses were conducted using two-sided tests, and a P-value < 0.05 was considered statistically significant. For missing data, when the missing proportion of a sample is less than 5% and missing completely at random, mean imputation is used; when the missing proportion is greater than 5% but less than 20%, multiple imputation is applied. If the missing proportion exceeds 20%, the sample is directly excluded.

## 3 Results

### 3.1 Baseline characteristics between the NSSI and non-NSSI groups

Overall, the mean age of the study sample was 15.4 years (± 1.0), with 248 males (42.18%) and 340 females (57.82%). Junior middle school students accounted for 58.84% of the sample, while senior high school students accounted for 41.16%. Approximately 73.13% of the participants were living with both parents, and 38.44% were only children. Compared with the non-NSSI group, the NSSI group had lower proportions of living with both parents and lower levels of peer support, while exhibiting higher levels of suicidal behaviors, depression, anxiety, internet addiction, sleep problems, and behavioral and emotional difficulties. No significant differences were observed between the two groups in demographic variables (**Table 1**). The attrition rate in this study was 8.84%. There were no significant differences between completers and non-completers in age, gender, grade level, parental education level, or only-child status, suggesting a low risk of systematic bias due to attrition (**Supplementary Table 1**).

**Table 1:** Comparison of baseline characteristics between the NSSI and non-NSSI groups.

Variables	TOTAL (n=588)	Non-NSSI (n=448)	NSSI (n=140)	P-value
**Age**	15.4 ( ± 1.0)	15.3( ± 1.0)	15.5 ( ± 0.9)	0.531
**Gender**				0.373
Male	248 (42.18%)	194 (43.3%)	54 (38.57%)	
Female	340 (57.82%)	254 (56.7%)	86 (61.43%)	
**Grade level**				0.052
Junior middle school	346 (58.84%)	274 (61.16%)	72 (51.43%)	
Senior high school	242 (41.16%)	174 (38.84%)	68 (48.57%)	
**Father education level**				0.161
Junior middle school and below	270 (45.92%)	201 (44.87%)	69 (49.29%)	
Senior high school or technical secondary school	219 (37.24%)	176 (39.29%)	43 (30.71%)	
Junior college and above	99 (16.84%)	71 (15.85%)	28 (20%)	
**Mother education level**				0.407
Junior middle school and below	318 (54.08%)	238 (53.12%)	80 (57.14%)	
Senior high school or technical secondary school	182 (30.95%)	145 (32.37%)	37 (26.43%)	
Junior college and above	88 (14.97%)	65 (14.51%)	23 (16.43%)	
**Living with both parents**				< 0.001
Yes	430 (73.13%)	347 (77.46%)	83 (59.29%)	
No	158 (26.87%)	101 (22.54%)	57 (40.71%)	
**Only-child status**				0.463
Yes	226 (38.44%)	168 (37.5%)	58 (41.43%)	
No	362 (61.56%)	280 (62.5%)	82 (58.57%)	
**Suicidal ideation**				< 0.001
Yes	42 (7.14%)	22 (4.91%)	20 (14.29%)	
No	546 (92.86%)	426 (95.09%)	120 (85.71%)	
**Suicide plan**				< 0.001
Yes	34 (5.78%)	12 (2.68%)	22 (15.71%)	
No	554 (94.22%)	436 (97.32%)	118 (84.29%)	
**Suicide attempt**				< 0.001
Yes	14 (2.38%)	1 (0.22%)	13 (9.29%)	
No	574 (97.62%)	447 (99.78%)	127 (90.71%)	
**Center for Epidemiologic Studies Depression (CES-D)**	15 (8-22)	14 (8-20)	16 (10-22)	0.025
**Screen for Child Anxiety Related Emotional Disorders (SCARED)**	25 (12-40)	24 (12-38)	27 (15-40)	0.017
**Young Internet Addiction Test (IAT)**	42 (30-52)	41 (30-49)	43 (30-52)	0.021
**Pittsburgh Sleep Quality Index (PSQI)**	6 (3-8)	5 (3-8)	6 (3-8)	0.009
**Bullying victimization**				< 0.001
Yes	78 (13.27%)	40 (8.93%)	38 (27.14%)	
No	510 (86.73%)	408 (91.07%)	102 (72.86%)	
**Multidimensional Scale of Perceived Social Support(MSPSS)(Friends subscale)**	17 (12-22)	18 (12-22)	16 (12-22)	0.007
**Strengths and Difficulties Questionnaire (SDQ)**	14 (10-18)	14 (10-18)	15 (10-18)	0.006
**Truancy**				0.060
Yes	27 (4.59%)	16 (3.57%)	11 (7.86%)	
No	561 (95.41%)	432 (96.43%)	129 (92.14%)	

### 3.2 Performance of four machine learning models for predicting NSSI at T1, T2, and T3

**Figure 1:**
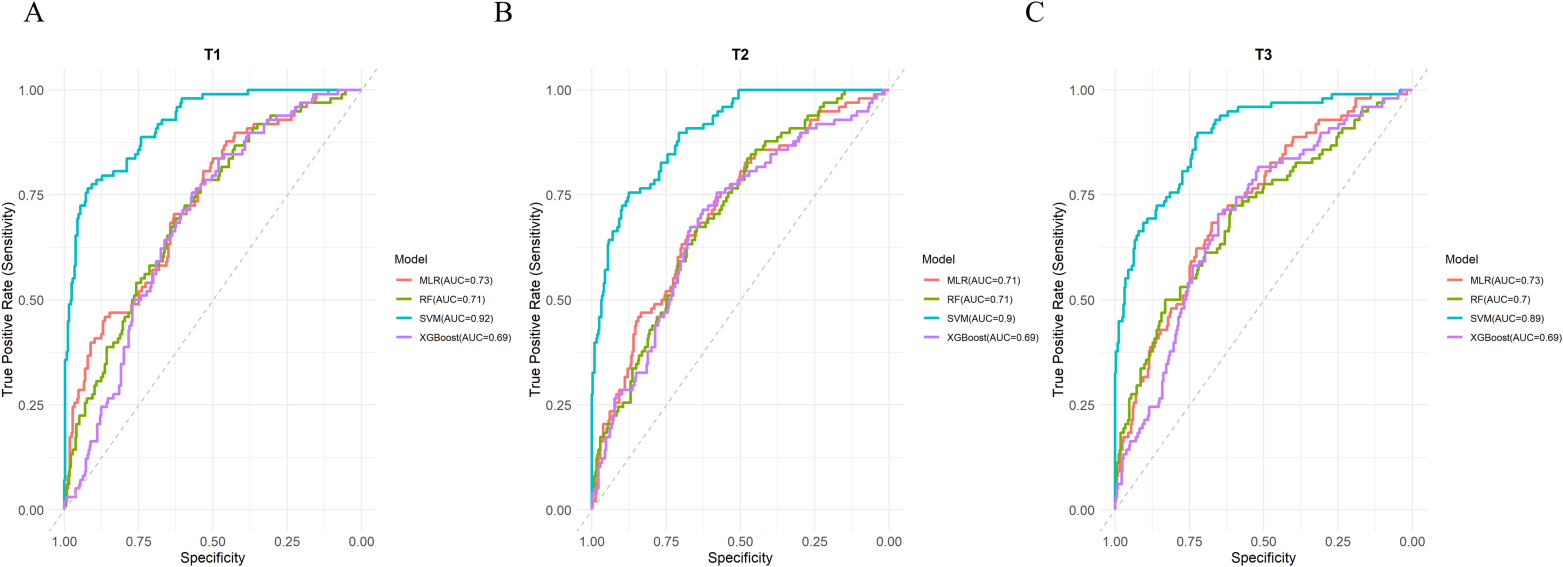
**(A)** ROC curves of four machine learning models at T1; **(B)** ROC curves of four machine learning models at T2; **(C)** ROC curves of four machine learning models at T3.

**Figure 2:**
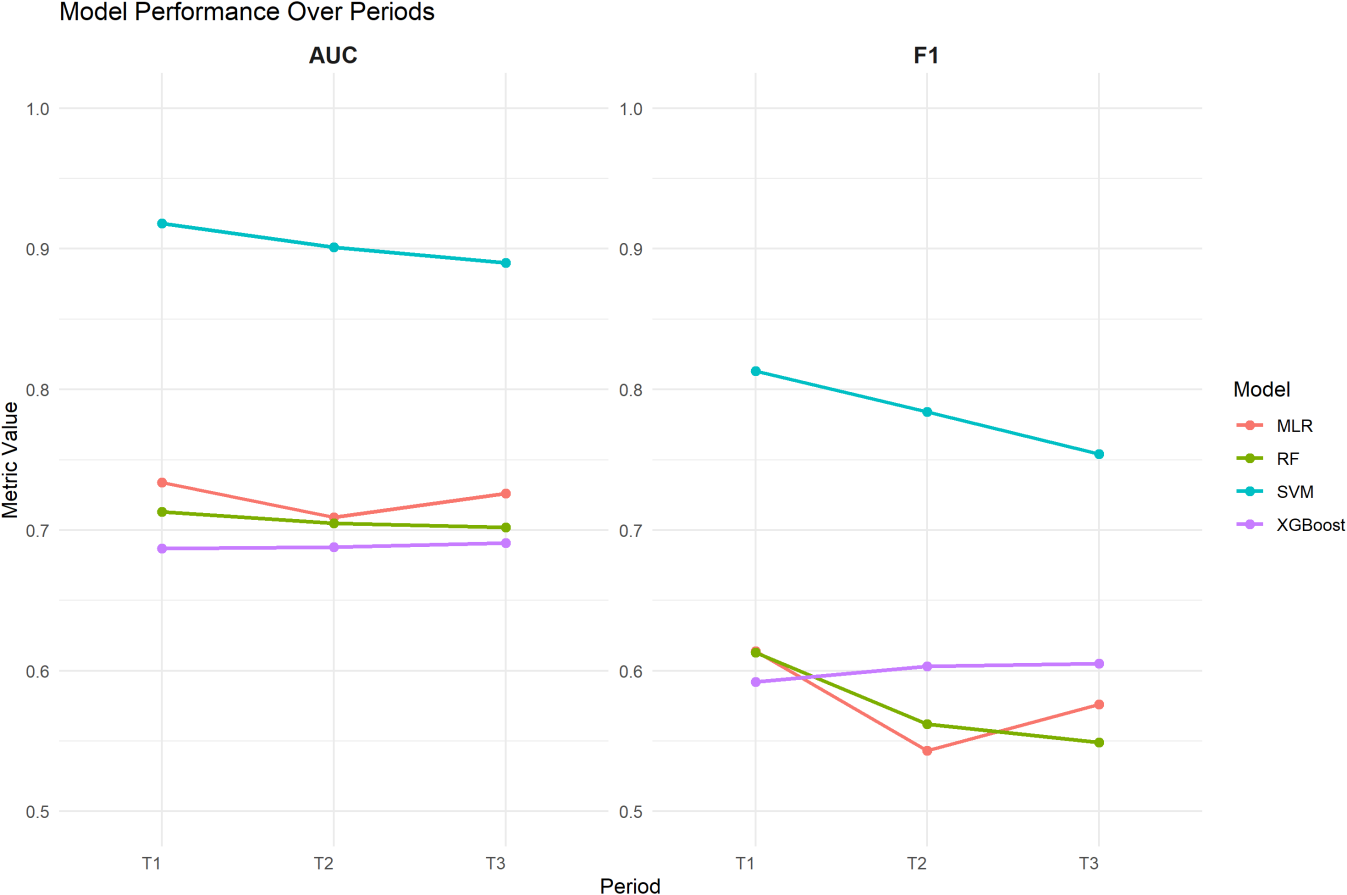
Trends in AUC values and F1 scores of four machine learning models over time.

**Figure 3:**
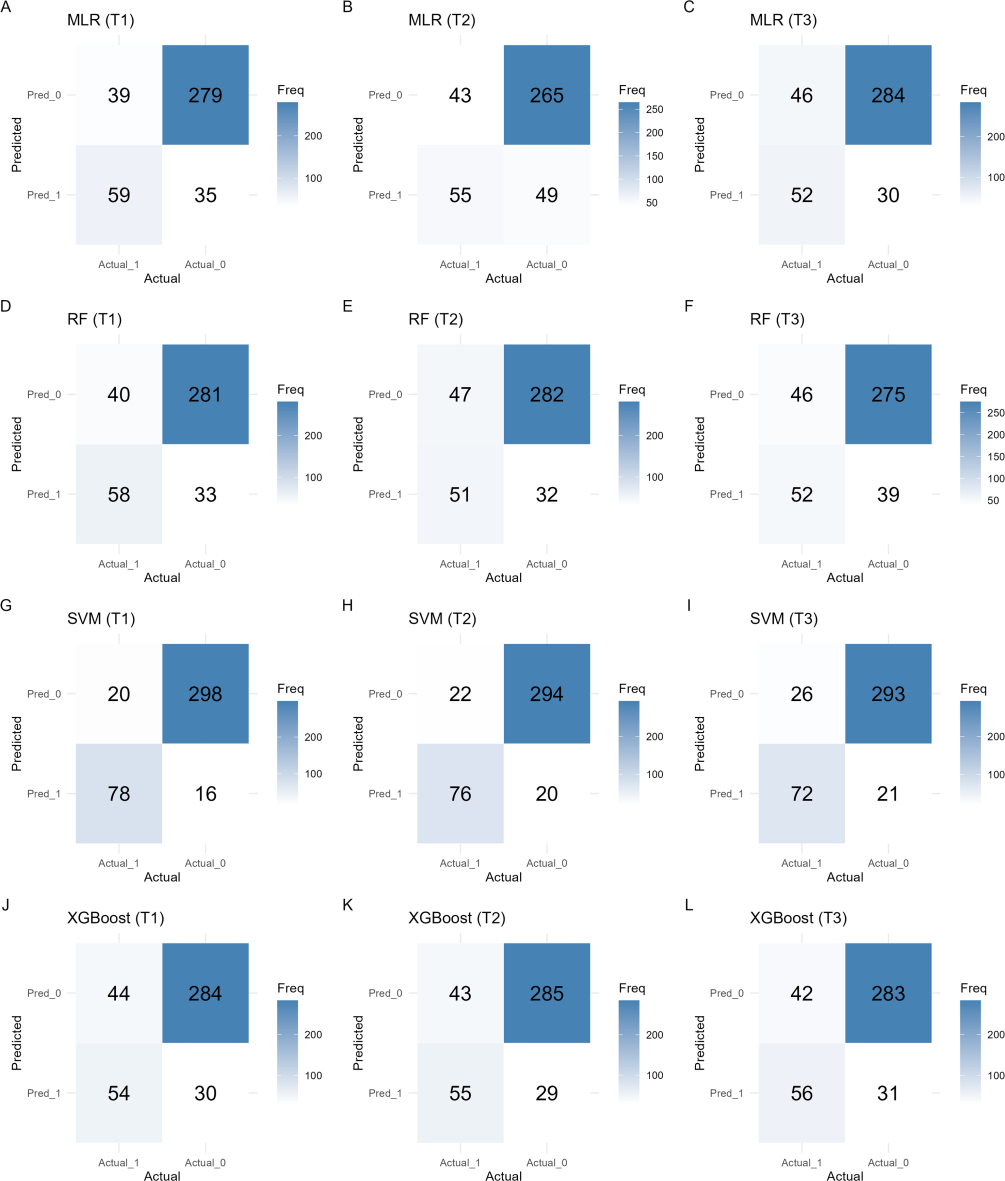
Visualization of the confusion matrix **(A–C)** MLR model for periods T1–T3; **(D–F)** RF model for periods T1–T3; **(G–I)** SVM model for periods T1–T3; **(J–L)** XGBoost model for periods T1–T3.

The significant factors identified in Section 3.1 were used as input features (n = 11), and multicollinearity analysis was performed. The results showed that all independent variables had VIF values well below the commonly accepted threshold (VIF < 5), indicating no multicollinearity among predictors (**Supplementary Table 2**). The SVM model achieved higher AUC values than the other three models at T1, T2, and T3 (**Figures 1A–C**), and the DeLong tests were statistically significant in all comparisons (P < 0.001) (**Supplementary Table 3**). In addition, its accuracy remained at a relatively high level (0.886–0.913), and its precision, recall, and F1-score were consistently superior to those of the other models (**Figure 2**). In contrast, the performance of the multivariate logistic regression (MLR), random forest (RF), and XGBoost models was relatively comparable. Among these models, the accuracy ranged from 0.776 to 0.826, and the AUC was approximately 0.687–0.734, indicating moderate discriminative ability. Confusion matrices for each model at T1, T2, and T3 are shown in **Figure 3**, illustrating the distribution of true positives, true negatives, false positives, and false negatives. Specifically, the MLR model showed a slightly higher AUC at T1 (0.734), while the RF model achieved better accuracy at T2 (0.808). From a temporal perspective, the performance of all models remained generally stable across T1, T2, and T3, with only minor fluctuations. Overall, the SVM model demonstrated the most stable and reliable performance in predicting NSSI across all three time points (**Table 2**).

**Table 2:** Performance of four machine learning models predicting NSSI at T1, T2, and T3.

		T1			T2			T3		
Model	Metric	Value	CI-lower	CI-upper	Value	CI-lower	CI-upper	Value	CI-lower	CI-upper
MLR	Accuracy	0.821	0.782	0.857	0.776	0.735	0.816	0.815	0.777	0.852
	Precision	0.628	0.531	0.725	0.529	0.434	0.626	0.633	0.526	0.737
	Recall	0.603	0.505	0.697	0.561	0.462	0.659	0.531	0.432	0.630
	F1	0.614	0.529	0.691	0.543	0.457	0.625	0.576	0.486	0.659
	AUC	0.734	0.674	0.795	0.709	0.647	0.770	0.726	0.664	0.789
RF	Accuracy	0.823	0.786	0.859	0.808	0.769	0.847	0.793	0.752	0.830
	Precision	0.638	0.537	0.734	0.614	0.511	0.718	0.571	0.469	0.674
	Recall	0.593	0.494	0.690	0.520	0.419	0.620	0.530	0.432	0.627
	F1	0.613	0.529	0.690	0.562	0.473	0.646	0.549	0.460	0.631
	AUC	0.713	0.669	0.756	0.705	0.675	0.736	0.702	0.667	0.738
SVM	Accuracy	0.913	0.833	0.991	0.898	0.831	0.953	0.886	0.860	0.910
	Precision	0.830	0.800	0.873	0.792	0.719	0.879	0.774	0.695	0.858
	Recall	0.796	0.725	0.856	0.776	0.728	0.830	0.735	0.628	0.802
	F1-score	0.813	0.752	0.894	0.784	0.717	0.854	0.754	0.705	0.808
	AUC	0.918	0.835	0.981	0.901	0.846	0.966	0.890	0.814	0.975
XGBoost	Accuracy	0.821	0.782	0.857	0.826	0.789	0.862	0.823	0.784	0.859
	Precision	0.643	0.542	0.744	0.655	0.554	0.758	0.644	0.541	0.744
	Recall	0.551	0.452	0.649	0.561	0.459	0.660	0.572	0.474	0.670
	F1-score	0.592	0.504	0.674	0.603	0.516	0.683	0.605	0.517	0.684
	AUC	0.687	0.664	0.710	0.688	0.663	0.713	0.691	0.664	0.718

Bold values indicate the variables included in the analysis of this study.

### 3.3 Visualization analysis of the SVM model

**Figure 4:**
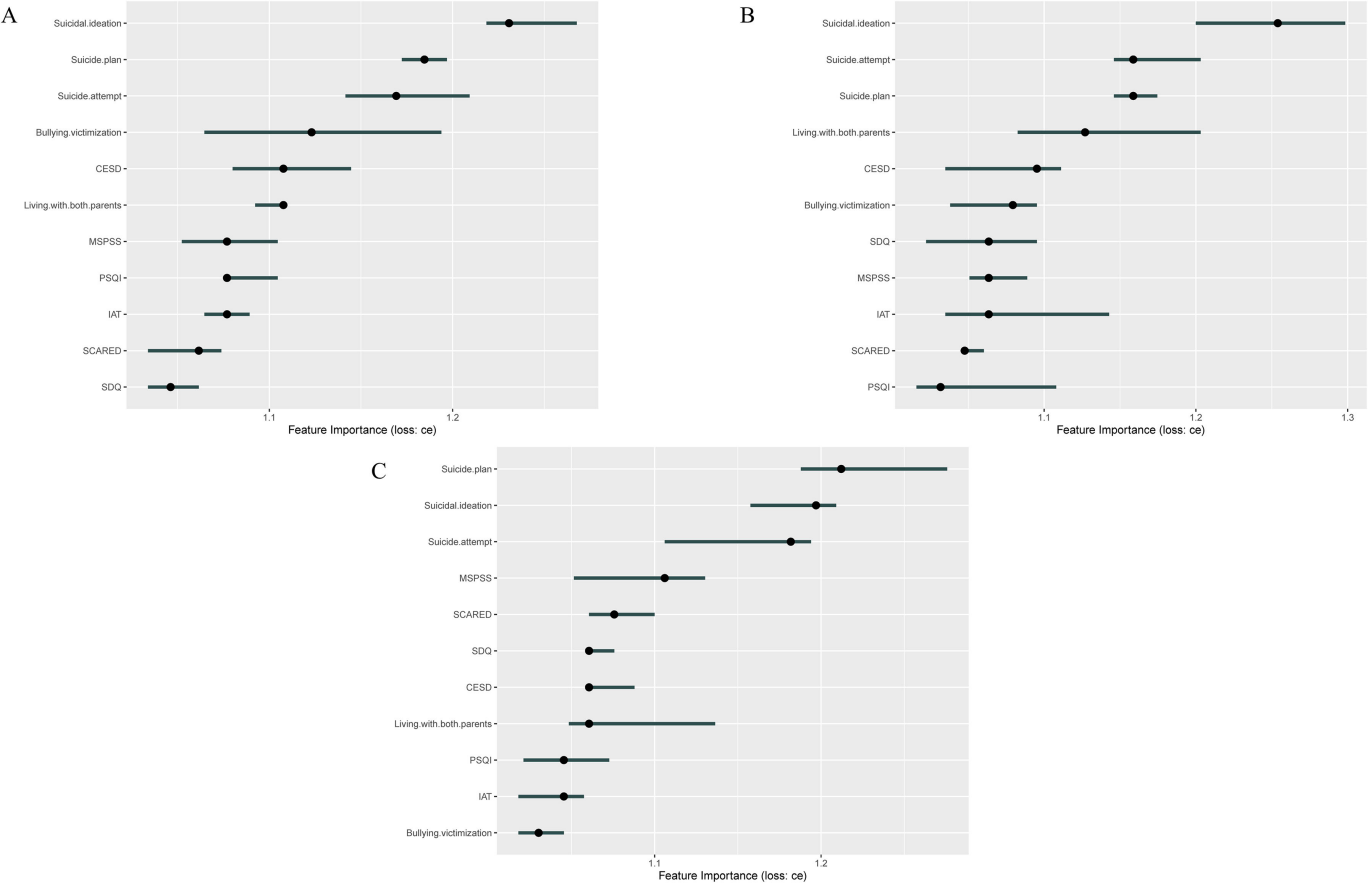
**(A)** SHAP visualization analysis of the SVM model at T1; **(B)** SHAP visualization analysis of the SVM model at T2; **(C)** SHAP visualization analysis of the SVM model at T3.

Based on the above analyses, the SVM model showed the best performance; therefore, SHAP visualization analysis was conducted for this model. The results indicated that suicide-related behaviors consistently ranked as the most important predictive feature across all follow-up periods. Experiences of school bullying, depression scores, and behavioral and emotional difficulty scores were also among the top predictive features (**Figures 4A–C**).

### 3.4 Internal validation

**Figure 5:**
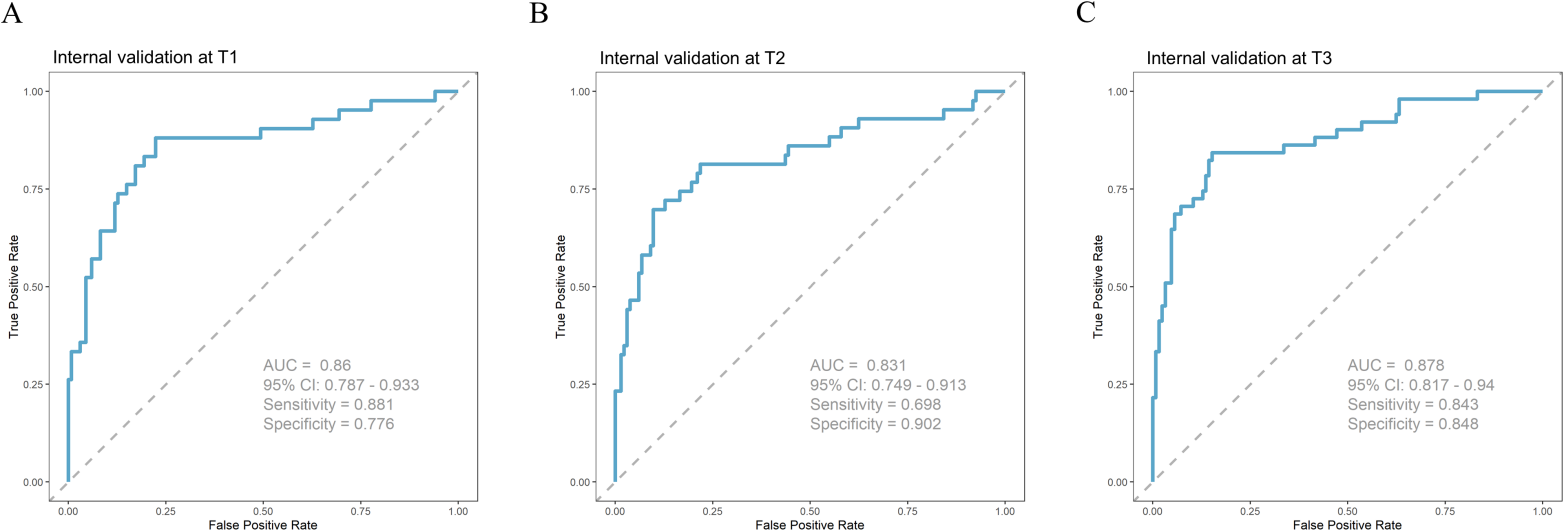
**(A)** Internal validation of the SVM model at T1; **(B)** Internal validation of the SVM model at T2; **(C)** Internal validation of the SVM model at T3.

Finally, we internally validated the SVM model on the test set. The results showed that the AUC values at T1–T3 were all higher than 0.83, accuracy was above 0.84, precision exceeded 0.74, recall was above 0.52, and F1-score was higher than 0.62, indicating an overall high level of performance (**Figures 5A–C****;** **Table 3**).

**Table 3:** Internal validation performance of the SVM model.

Metric	T1	T2	T3
Accuracy	0.852	0.841	0.869
Precision	0.786	0.742	0.838
Recall	0.524	0.535	0.646
F1-score	0.629	0.622	0.729

## 4 Discussion

This study found that the SVM model performed best in predicting NSSI among adolescents aged 12–18 years. Based on this model, SHAP visualization analysis indicated that suicide-related ideation and behaviors, experiences of school bullying, depressive status, and behavioral and emotional difficulties were important risk factors for NSSI.

Our study showed that suicide-related ideation and behaviors are important risk factors for the occurrence of NSSI, which is consistent with previous studies (28). A study conducted in Hong Kong indicated that NSSI often co-occurs with suicidal behaviors, and highly injurious methods of self-harm, frequent alcohol consumption, and sexual experience are important screening indicators of suicide risk among adolescents with NSSI (29). These studies collectively suggest that when adolescents experience suicidal ideation, they are often accompanied by strong feelings of depression or hopelessness. Some adolescents may engage in self-injurious behaviors to relieve negative emotions or divert emotional attention. Suicidal behaviors and NSSI share certain behavioral similarities in that both involve harm to the body. When adolescents have suicidal thoughts, it may indicate a lower psychological threshold for pain and reduced psychological barriers to self-harm, making them more likely to adopt NSSI, which has a relatively lower lethality risk, as a coping strategy.

In addition, experiences of bullying are also one of the risk factors for NSSI among adolescents, which is consistent with previous studies (30). Esposito et al. found that any experience of bullying, whether as a victim or perpetrator, increases the risk of NSSI. When victims of school bullying simultaneously experience peer rejection, their risk of engaging in NSSI further increases (31). A study by Ashley et al. showed that for adolescents who have experienced school bullying and exhibit depressive symptoms, stronger connections with family and school can reduce experiences of bullying and depressive symptoms, thereby indirectly lowering the likelihood of NSSI (32). Therefore, certain intervention strategies can be implemented to reduce the incidence of NSSI among victims of school bullying, such as improving communication and understanding between parents and children to enhance family connectedness, which can directly alleviate psychological stress in adolescents, as well as strengthening students’ sense of school belonging and creating a safe and supportive school environment.

The strength of this study lies in the use of longitudinal data analysis. Previous machine learning studies mainly analyzed the influence of psychological and behavioral factors on NSSI at a single time point, whereas the present study integrated follow-up data from multiple time points with six-month intervals between each assessment. This interval is consistent with the academic and daily life rhythms of middle and high school students. Such relatively short intervals not only allow the capture of potential psychological and behavioral changes among adolescents across different academic semesters but also help reduce information bias that may arise from excessively long follow-up periods, thereby enabling a more accurate assessment of the relationship between various risk factors and the occurrence of NSSI. By using baseline (T1) variables to predict NSSI outcomes at T1, T2, and T3, the study allows for the evaluation of model stability and generalizability across time, thereby providing more temporally extended evidence for NSSI risk prediction.

This study also has several limitations. First, as a retrospective study, there may be a certain degree of selection bias in the data. Second, most of the study variables were obtained through questionnaire surveys. Although questionnaires are the primary method for studying NSSI, they still rely on self-reported data, which may be influenced by recall bias or social desirability bias, thereby affecting the accuracy of the information. Third, we did not fully include other potential influencing factors, such as academic pressure and traumatic experiences. In addition, the sample was drawn from a specific adolescent population, which may limit the generalizability of the findings to different cultural contexts or clinical populations. The study outcome was defined as the occurrence of NSSI within the past year, whereas the follow-up interval was 6 months. This results in a certain degree of overlap between the prediction window and the outcome window, which may consequently affect model performance. Future studies could adopt multi-center prospective designs and include a broader range of influencing factors to further improve the reliability and generalizability of the results.

## Data Availability

The raw data supporting the conclusions of this article will be made available by the authors, without undue reservation.
