# Supplementary material for: Development and validation of a machine learning–based risk prediction model for non-suicidal self-injury in adolescents
Source: Front Psychiatry. 2026 May 13;17:1837161. doi: 10.3389/fpsyt.2026.1837161 (PMC13212310; doi:10.3389/fpsyt.2026.1837161)
Supplement: Supplementary file 1 [file Table1.docx]

| **Supplementary Table. 1 Comparison of characteristics between participants who completed follow-up and those lost to follow-up** | | | |
| --- | --- | --- | --- |
|  | **Included (n=588)** | **Excluded (n=52)** | **P-value** |
| **Age** | 15.4  (±1.0) | 15.5  (±1.1) | 0.282 |
| **Gender** |  |  | 0.4975841 |
| Male | 248 (42.18%) | 25 (48.08%) |  |
| Female | 340 (57.82%) | 27 (51.92%) |  |
| **Grade level** |  |  | 0.8167679 |
| Junior middle school | 346 (58.84%) | 32 (61.54%) |  |
| Senior high school | 242 (41.16%) | 20 (38.46%) |  |
| **Father education level** |  |  | 0.3186 |
| Junior middle school and below | 270 (45.92%) | 27 (51.92%) |  |
| Senior high school or technical secondary school | 219 (37.24%) | 14 (26.92%) |  |
| Junior college and above | 99 (16.84%) | 11 (21.15%) |  |
| **Mother education level** |  |  | 0.9544 |
| Junior middle school and below | 318 (54.08%) | 27 (51.92%) |  |
| Senior high school or technical secondary school | 182 (30.95%) | 17 (32.69%) |  |
| Junior college and above | 88 (14.97%) | 8 (15.38%) |  |
| **Living with both parents** |  |  | 0.1800979 |
| Yes | 430 (73.13%) | 43 (82.69%) |  |
| No | 158 (26.87%) | 9 (17.31%) |  |
| **Only-child status** |  |  | 0.6930738 |
| Yes | 226 (38.44%) | 18 (34.62%) |  |
| No | 362 (61.56%) | 34 (65.38%) |  |

| **Supplementary Table. 2 Variance inflation factors (VIF) for all independent variables** | |
| --- | --- |
| **Variables** | **VIF** |
| Living with both parents | 1.029 |
| Suicidal ideation | 1.024 |
| Suicide plan | 1.028 |
| Suicide attempt | 1.005 |
| CESD | 1.026 |
| SCARED | 1.022 |
| IAT | 1.014 |
| PSQI | 1.023 |
| Bullying victimization | 1.018 |
| MSPSS | 1.014 |
| SDQ | 1.031 |

| **Supplementary Table. 3 Results of the DeLong test** | | | |
| --- | --- | --- | --- |
| **Comparison** | **T1 p-value** | **T2 p-value** | **T3 p-value** |
| SVM vs MLR | < 0.001 | < 0.001 | < 0.001 |
| SVM vs RF | < 0.001 | < 0.001 | < 0.001 |
| SVM vs XGBoost | < 0.001 | < 0.001 | < 0.001 |
